# Establishment and analysis of the prediction model for the prognosis of children with sepsis based on pSOFA score

**DOI:** 10.12669/pjms.41.4.10001

**Published:** 2025-04

**Authors:** Fang Guo, Yi Qu, Lei Kang, Meixian Xu, Li Ma, Wenhui Li, Dianping You

**Affiliations:** 1 Fang Guo Department of Infectious Diseases, Hebei Children’s Hospital, Shijiazhuang 050000, Hebei, China; 2 Yi Qu Department of Scientific research, Hebei Children’s Hospital, Shijiazhuang 050000, Hebei, China; 3 Lei Kang Department of Pediatric Intensive Care Unit, Hebei Children’s Hospital, Shijiazhuang 050000, Hebei, China; 4 Meixian Xu Department of Pediatric Intensive Care Unit, Hebei Children’s Hospital, Shijiazhuang 050000, Hebei, China; 5 Li Ma Department of Neonatology, Hebei Children’s Hospital, Shijiazhuang 050000, Hebei, China; 6 Wenhui Li Department of Infectious Diseases, Hebei Children’s Hospital, Shijiazhuang 050000, Hebei, China; 7 Dianping You Pediatric Clinical Research Centre, Hebei Children’s Hospital, Shijiazhuang 050000, Hebei, China

**Keywords:** Febrile neutropenia, Pediatric sequential organ failure assessment (pSOFA), Sepsis, Systemic vascular resistance index (SVRI)

## Abstract

**Objective:**

To investigate the prognostic risk factors of sepsis in children diagnosed based on the pediatric sequential organ failure assessment (pSOFA) scoring system, and to establish and evaluate related prediction model.

**Methods:**

This was a retrospective study. Two hundred and seventy three children with sepsis admitted to Hebei Children’s Hospital from January 1, 2019 to December 31, 2022 were divided into survival group and mortality group according to the prognosis. Multivariate Logisitic regression was used to analyze the independent risk factors affecting the prognosis of septic children, and a predictive model was established to analyze the predictive value of the model on the prognosis of septic children.

**Results:**

The results of multivariate logistic regression analysis showed that systemic vascular resistance index (SVRI) <1143, febrile neutropenia (FN) and pSOFA score were independent risk factors for sepsis-related death in children. This equation of 5.140+2.069 × (SVRI<1143) + 1.718 × FN+0.290 × pSOFA score indicates that, the area under the ROC curve of the prediction model is greater than that of SVRI<1143, FN, and pSOFA score alone, and the sensitivity and specificity of the model were 81.5% and 84.5%, respectively.

**Conclusion:**

The prediction model has a good clinical predictive value and has practical significance for the prognosis evaluation and treatment guidance.

## INTRODUCTION

Sepsis is a life-threatening organ dysfunction resulting from the dysregulated host response to infection^1^, which poses a non-negligible threat to the survival of children. Approximately 20 million cases of pediatric sepsis were reported in the world per year, with 2.9 million sepsis-related deaths among children younger than five years old, accounting for 19.7% of the global deaths.^2^ At present, the diagnostic criteria for pediatric sepsis in our country are mostly based on the “International pediatric sepsis consensus conference (2005)”^3^ and the pediatric part of the “Surviving sepsis campaign: international guidelines for management of severe sepsis and septic shock: 2012”.^4^ The criteria are too heterogeneous and in lack of quantitative indicators, which restricted the universal application in pediatric emergency departments and general wards.

In 2016, the European Society of Intensive Care Medicine (ESICM) adopted the sequential organ failure assessment (SOFA) score ≥2 as the core criteria of sepsis 3.0^1^, which greatly simplified the diagnostic process in adult sepsis. Pediatric sequential organ failure assessment (pSOFA) was proposed by Matics^5^ in 2017. Subsequent studies have shown that pSOFA has a good ability to predict death in children with sepsis^6^, but it has also been found that the mortality of septic children who suffered from chronic diseases, hematological diseases, severe malnutrition and mechanical ventilation was increased^7^, while the pSOFA score ignored the above clinical factors. Knowing that using pSOFA alone as an assessment method for the prognosis might affect the early identification and precise treatment, we conducted this study based on the sepsis 3.0 and the pSOFA proposed by Matics^5^ to explore the independent risk factors for death, and established predictive model on the prognosis of children with sepsis.

## METHODS

A retrospective analysis of 273 children with sepsis admitted to the pediatric intensive care unit (PICU) in Hebei Children’s Hospital from January 1, 2019 to December 31, 2022 was performed. All subjects were divided into survival group(n=219) and Mortality Group (n=54) according to the 30-day clinical outcome.

### Ethical Approval:

The study was approved by the Institutional Ethics Committee of Hebei Children’s Hospital (No.: 202103; date: March 19, 2021), and written informed consents were provided by the legal guardians of enrolled children.

### Inclusion criteria:

- Meeting the criteria for Sepsis3.0, Sepsis3.0= infection +SOFA≥2.^1^
- Adopting pSOFA score proposed by Matics.^5^
- Age: 29 days,18 years (corrected gestational age of preterm infants >41 weeks).

### Exclusion criteria:

- Those who died within 24 hours after admission.
- Combined primary immunodeficiency or inherited metabolic diseases.
- Underwent accidental injury (poisoning, foreign body inhalation, falling from height, car accident injury, impact injury, etc.).

### Data Collection:

The clinical manifestations of patients and laboratory data including age and gender, primary disease, length of stay in PICU, therapeutic measures, the presence or absence of febrile neutropenia (FN), pSOFA score (the highest score of pSOFA at one hour, six hours and 24 hours after admission), focus of infection, prognosis, white blood cells (WBC), absolute neutrophil count (ANC), hemoglobin (Hb), platelets (PLT), International Normalized Ratio (INR), D-Dimer (DD), C-reactive protein (CRP), procalcitonin (PCT), blood gas analysis, CD4+T cells, Immunoglobulin G (IgG), and so forth were collected from the inpatient medical records.

Hemodynamic data including cardiac index (CI), stroke volume variation (SVV), and systemic vascular resistance index (SVRI) were obtained by non-invasive cardiac output monitoring system (NICaS)^8^, which can detect SVRI by Friner-man and Tsoglin algorithms.^9^ These data were conducted at admission or within 24 h after admission.

### Statistical analysis:

SPSS 24.0 software was used for analysis; the confidence interval was 95%. After examining the normality of distribution, quantitative variables were represented as the mean ± standard deviation (SD) or the median (P25, P75). Comparisons between two groups were analyzed with independent t-test or Mann-Whitney U test, where appropriate. Chi-square test or Fisher’s exact test was used for comparison of counting data, univariable and multivariable logistic regression analysis were used to identify the associations of variables with mortality in septic children.

The receiver-operator curve (ROC) was drawn to evaluate the predictive value of variables for mortality, Nomogram was introduced and predictive model was established using R4.0.3 software, the Bootstrap method was applied to internally sample 1000 times for verification, the calibration curve to evaluate consistency, the Hosmer-Lemeshow test to judge the goodness of fit, and the decision curve to assess the clinical effect. *P*< 0.05 was considered statistically significant.

## RESULTS

A total of 273 septic children with a median age of 26(8, 82) months were enrolled in the study, including 150 boys and 123 girls. According to the prognosis, 219 cases were in the survival group and 54 cases were in the Mortality Group, which means that the mortality was 19.8%. No significant differences were observed in age, sex, primary disease, Hb, CRP, blood glucose, blood calcium, INR, DD, lactic acid, CD8+, NK, IgG, CI, SVV, and the usage of special grade antibiotics, hemopurification, and glucocorticoid between the two groups. The pSOFA score, WBC, ANC, PCT and presence of FN in Mortality Group were significantly higher than those of survival group (*p* < 0.05), SVRI and CD4+T were significantly lower than those of survival group (*p*< 0.05). The demographic characteristics and laboratory parameters of two groups are presented in Table-I.

**Table-I:** Comparison of general information between survival group and Mortality Group.

Parameters	Survival group(n=219)	Mortality Group(n=54)	t/χ^2^ /Z	P
Age(month)	24.0(7.0,81.0)	47.0(17.5,83.3)	Z=-1.923	0.054
Boys [n(%)]	116(53.0)	34(63.0)	χ^2^=1.748	0.186
pSOFA	5.0(4.0,8.0)	10.0(7.0,14.0)	Z=-6.337	0.000
Leukemia [n(%)]	21(9.6)	10(18.5)	χ^2^=3.431	0.064
Kidney disease[n(%)]	14(6.4)	5(9.3)	χ^2^=0.196	0.658
CI[L/(min·m^2^)]	3.5±1.3	3.3±1.2	t=1.175	0.241
SVV (%)	14.5±3.1	14.0±3.1	t=1.128	0.260
SVRI (dyn·sec/cm^5^/m^2^)	1227.7±360.3	927.9±242.2	t=6.220	0.000
SVRI<1143[n(%)]	77(35.2)	42(77.8)	χ^2^=31.998	0.000
WBC (×10^9^/L)	13.8(4.1,22.2)	4.2(0.5,13.7)	Z=-3.865	0.000
ANC (×10^9^/L)	7.3(1.9,14.1)	0.7(0.1,9.8)	Z=-4.199	0.000
FN [n(%)]	37(16.9)	29(53.7)	χ^2^=32.018	0.000
Hb(g/L)	99.1±21.2	93.3±23.9	t=1.763	0.079
blood glucose (mmol/L)	5.9(4.6,7.7)	6.5(5.1,7.7)	Z=-1.041	0.298
blood calcium (mmol/L)	1.6(1.2,2.1)	1.4(1.0,1.8)	Z=-1.916	0.055
CRP(mg/L)	76.2(31.8,147.0)	102.4(56.2,165.4)	Z=-1.862	0.063
PCT(μg/L)	8.6(2.1,18.3)	10.1(5.0,44.3)	Z=-2.033	0.042
INR	3.6(2.5,4.2)	3.1(2.3,4.3)	Z=-0.959	0.338
DD(mg/L)	4.0(1.8,18.1)	6.7(1.4,24.4)	Z=-0.780	0.435
lactic acid(mmol/L)	5.2±1.6	5.3±1.7	t=-0.879	0.380
CD4+T(μ/L)	260.9(163.0,355.0)	230.0(138.8,303.6)	Z=-2.003	0.045
CD8+T(μ/L)	117.0(80.0,183.0)	98.0(62.3,150.2)	Z=-1.691	0.091
NK(μ/L)	127.1±41.8	124.6±47.8	t=-0.648	0.518
IgG(g/L)	9.6(7.4,12.3)	8.8(7.0,10.4)	Z=-1.680	0.093
Mechanical ventilation [n(%)]	106(48.4)	34(63.0)	χ^2^=3.676	0.055
Special grade antibiotics [n(%)]	127(58.0)	39(72.2)	χ^2^=3.681	0.055
Hemopurification [n(%)]	165(75.3)	44(81.5)	χ^2^=0.910	0.340
Glucocorticoid [n(%)]	82(37.4)	24(44.4)	χ^2^=0.894	0.344
Length of hospitalization(d)	16.0(11.0,21.6)	3.0(1.0,5.0)	Z=-8.656	0.000

Coordinates of ROC curve may determine the cut- off value of SVRI which meant as death threshold and it obtained SVRI threshold value was 1143 with a sensitivity value of 77.8% and a specificity value of 64.8%. Then, multivariable logistic regression analyses were performed to assess associations of these parameters with mortality in septic children. The results indicated that SVRI<1143 (OR=7.918, 95%CI: 3.373~18.588), FN (OR=5.575, 95%CI: 2.530~12.285) and pSOFA score (OR=1.337, 95%CI: 1.201~1.489) were independent risk factors for sepsis-related death in children (Table-II).

**Table-II:** Multivariate logistic regression for prognosis in children with sepsis.

Parameters	OR	95%CI	P
SVRI<1143	7.918	3.373~18.588	0.000
pSOFA	1.337	1.201~1.489	0.000
FN	5.575	2.530~12.285	0.000
PCT	0.999	0.993~1.005	0.775
CD4+T	0.999	0.996~1.002	0.444

**Fig.1:**
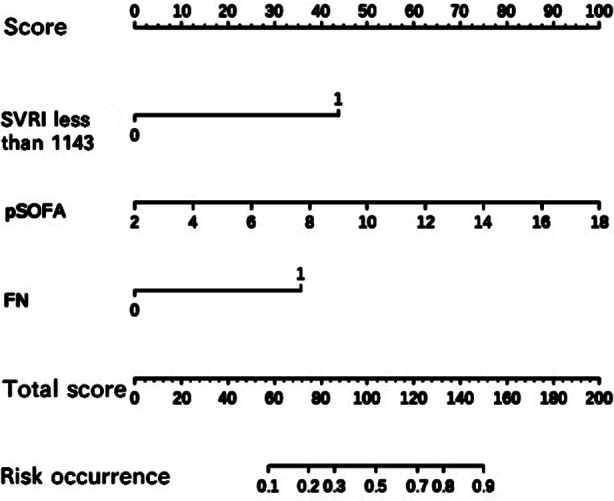
Nomogram of prognosis in children with sepsis.

**Fig.2:**
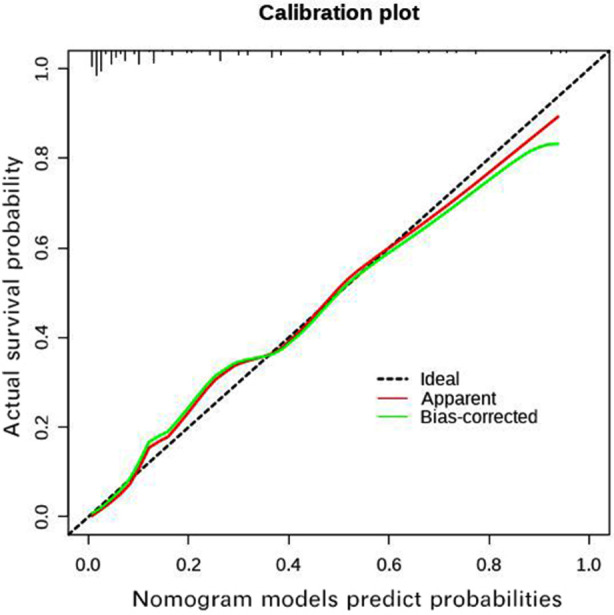
Calibration curves of the nomogram in children with sepsis.

**Fig.3:**
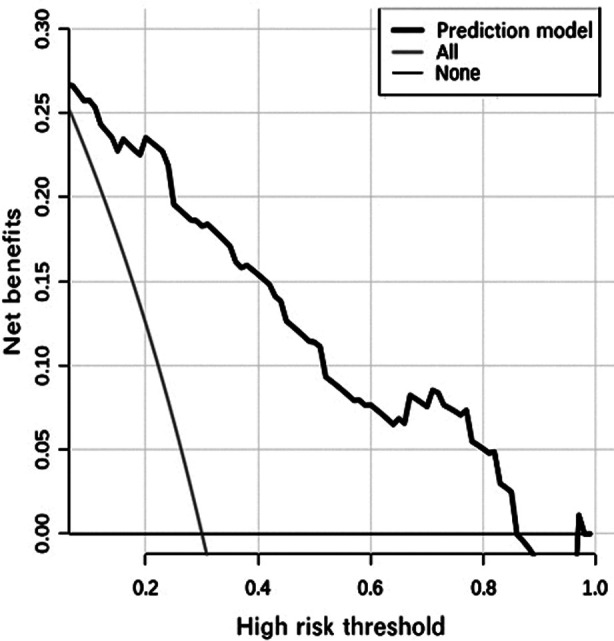
The decision curve of the nomogram in children with sepsis.

By incorporating these independent risk factors into the nomogram (Fig.1), we obtain the model equations=-5.140+2.069×(SVRI<1143)+1.718×FN+0.290×pSOFA score. The Bootstrap method was used to internally sample 1000 times for verification, and the calibration curve judged that the predicted value was more consistent with the actual value (Fig.2). Hosmer-Lemeshow test was used to determine the validity of fit, which showed well calibration ability (c^2^=6.150, P=0.630) of the model. Decision curve analysis (Fig.3) showed that high-risk thresholds between 0.02 and 0.97 had higher net benefits, suggesting that the predictive model has higher clinical value.

**Fig.4:**
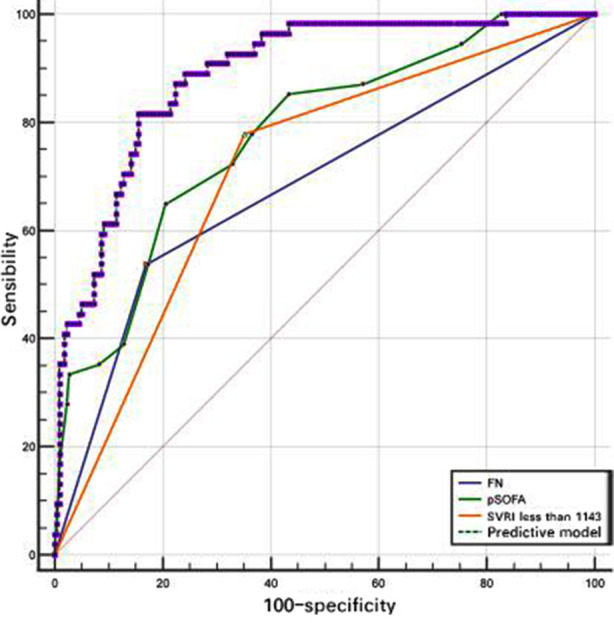
Comparison of the prognostic value between independent factor and model.

The ROC were plotted to evaluate the predictive value of prediction model, SVRI<1143, FN, and pSOFA score for mortality respectively in children with sepsis. The pSOFA score as continuous variable was converted to a categorical variable and its cut-off value was 8.5. Only prediction model had the best efficacy to predict death, the area under the ROC curve for prediction model (0.889) was greater than that of SVRI<1143 (0.713), FN (0.684), and pSOFA score (0.777) alone and had the best combination (Fig.4), with a sensitivity 81.5% and a specificity 84.5% (Table-III).

**Table-III:** Comparison of different indicators for predictive value.

Parameters	AUC	95%CI		Cut- off values		Sensitivity (%)		Specificity (%)		Comparison with model			
											Z		P
SVRI<1143	0.713	0.655~0.766		—		77.8		64.8		5.973		<0.0001	
pSOFA	0.777	0.723~0.825		8.5		64.8		79.5		3.847		0.0001	
FN	0.684	0.665~0.739		—		53.7		83.1		5.542		<0.0001	
Prediction model	0.889	0.846~0.924		0.150		81.5		84.5		—		—	

## DISCUSSION

Sepsis is a major threat to children worldwide, and the morbidity and mortality of sepsis in our country are much higher than those in North America and European countries.^10^ The 30 days mortality of children with sepsis in this study was higher than that reported in other children’s hospitals of China^11^, which may be related to the different diagnostic criteria of the enrolled subjects. The latter was based on the 2012 guideline, in which the children that met the inflammatory indicators but not complicated with organ dysfunction were also diagnosed as sepsis, who survived well after anti-infection treatment, but our study excluded this group. In our study, Sepsis 3.0 was used as the diagnostic criterion, the results showed that taking pSOFA score 8.5 as the cut-off value, the area under the curves (AUC) for pSOFA score was 0.777 with a sensitivity 64.8% and a specificity 79.5%, and pSOFA score was associated with a statistically significant increased risk of death, which was consistent with the previous studies.^12^

Only organ dysfunction secondary to infection can be classified as sepsis. Using pSOFA score which lacks infection indicators as an assessment alone for sepsis may lead to excessive interventions. Therefore, we included variables such as PCT, CRP, WBC, PLT and FN to explore the efficacy of pSOFA score combined with other parameters in predicting death. The AUC for prediction model was 0.889 with 95% confidence interval (0.846~0.924), and the cut off value for prediction model was 0.150, with a sensitivity 81.5% and a specificity 84.5%, and had the best combination compared with pSOFA score alone, suggesting that the model can better predict death.

Septic shock occurred when the inflammatory response worsened excessively, resulting in microcirculation disturbance, organ hypoperfusion and cardiovascular dysfunction. Previous studies have shown that the mortality of septic shock was about six times higher than that of patients with sepsis.^13^ Due to the lack of specific quantitative indicators in the early stage of shock, effective interventions were often missed. Once blood pressure dropped, it indicated the decompensation stage with poor treatment response.

In this study, NICaS was performed to reflect the cardiovascular function by measuring hemodynamic indicators, and the results showed that the risk of death was significantly increased with SVRI < 1143, which was an independent risk factor for prognosis. SVRI represented cardiac afterload, and sustained vasoparesis was the physiological basis for reduced SVRI, indicating a higher risk of death for worse systemic vascular response to fluid resuscitation and vasoactive drugs.^14^ Contraction of vascular smooth muscle cells determined vascular tone, and vasoparalysis in children with sepsis was regulated by intrinsic and extrinsic mechanisms^15^:

- Endothelial secretions, such as nitric oxide, prostacyclin and endothelin;
- Endocrine hormones such as serotonin, bradykinin, and thrombin;
- Vasoactive metabolites such as acidosis, hypoxia, and hydrogen peroxide;
- Sympathetic and renin-angiotensin system. The vascular paralysis caused by the aforementioned mechanisms brought great challenges to the treatment of children with sepsis. Therefore, effective hemodynamic monitoring was helpful for early detection of low SVRI and corresponding interventions, which in turn would improve the prognosis.

We found that FN was an independent risk factor for death in children with sepsis, and the risk of death in septic children combined with FN increases by more than 5.5 times, the possible mechanisms are as follows:

- Obstruction in pathogen clearance: neutrophils are important phagocytes in innate immunity; they quickly arrive at the focus when infection strikes and triggers a flurry of activity, such as the release of reactive oxygen species, degranulation, formation of neutrophil extracellular traps and phagocytosis^16^,^17^, which promotes antibacterial defense mechanisms by capturing and subsequently killing bacteria. Therefore, a severe decline in their numbers can hinder the clearance of pathogens and lower the defense barrier;
- Immune dysregulation: neutrophils may assist in the activation of T lymphocytes through antigen presentation, and regulate dendritic cells, lymphocytes and natural killer cells through membrane molecules or secretion of specific cytokines.^18^ Children with FN have dysregulation of innate and adaptive immunity, which together with clearance disorders of infection can lead to aggravation of organ dysfunction or death. Previous studies^19^,^20^ have shown that myocardial ischemia-reperfusion injury, arteriovenous thrombosis, and sepsis-associated acute respiratory distress syndrome were related to neutrophil-related immune dysregulation.

### Limitations:

Firstly, the study was single-center retrospective study and the number of cases was limited with inevitable bias. Secondly, as this is a retrospective study, the expressions of important inflammatory cytokines in sepsis such as interleukin, tumor necrosis factor and interferon were not tested at that time for some patients, so we excluded these variables. Finally, for some reasons, there is no in-depth analysis of FN caused by different causes. Therefore, a larger multi-center prospective sample study is needed to avoid bias and these limitations.

## CONCLUSIONS

SVRI<1143, FN and pSOFA score are independent risk factors for sepsis-related death in children. The prediction model has a good clinical predictive value and has practical significance for the prognosis evaluation and treatment guidance.

### Authors’ Contributions:

**FG** and **LK:** Carried out the studies, participated in collecting data, and drafted the manuscript, were responsible and accountable for the accuracy or integrity of the work.

**YQ** and **MX:** Performed the statistical analysis and participated in its design.

**LM, WL** and **DY:** Participated in acquisition, analysis, or interpretation of data and drafted the manuscript. All authors read and approved the final manuscript.
